# Consensus guidelines for newborn screening, diagnosis and treatment of infantile Krabbe disease

**DOI:** 10.1186/s13023-018-0766-x

**Published:** 2018-02-01

**Authors:** Jennifer M. Kwon, Dietrich Matern, Joanne Kurtzberg, Lawrence Wrabetz, Michael H. Gelb, David A. Wenger, Can Ficicioglu, Amy T. Waldman, Barbara K. Burton, Patrick V. Hopkins, Joseph J. Orsini

**Affiliations:** 1 University of Rochester Medical Center, 601 Elmwood Avenue, Box 631, Rochester, NY 14642 USA; 2 Biochemical Genetics Laboratory, Mayo Clinic, 200 First Street SW, Rochester, MN 55905 USA; 3 Pediatric Blood and Marrow Transplant Program, Duke University Medical Center, 2400 Pratt Street, Durham, NC 27705 USA; 4 Hunter James Kelly Research Institute (HJKRI), University at Buffalo Jacobs School of Medicine and Biomedical Sciences, NYS Center of Excellence, 701 Ellicott St, Buffalo, NY 14203 USA; 5 Department of Chemistry and Biochemistry, University of Washington, Seattle, WA 98195 USA; 6 Sidney Kimmel Medical College, 1020 Locust St, Room 346, Philadelphia, PA 19107 USA; 7 The Children’s Hospital of Philadelphia, Division of Human Genetics and Metabolism, 3501 Civic Center Blvd., Philadelphia, PA 19104 USA; 8 The Children’s Hospital of Philadelphia, Leukodystrophy Center, Division of Neurology, 34th Street and Civic Center Boulevard, Philadelphia, PA 19104 USA; 9 Ann & Robert H. Lurie Children’s Hospital, 225 E. Chicago Avenue, Chicago, IL 60611 USA; 10 Newborn Screening Unit Missouri State Public Health Laboratory, 101 N. Chestnut St., PO Box 570, Jefferson City, MO 65102-0570 USA; 11 Wadsworth Center, New York State Department of Health, Newborn Screening Program, David Axelrod Institute, 120 New Scotland Ave., Albany, NY 12201 USA

**Keywords:** Krabbe disease, Newborn screening, Guidelines, Human stem cell transplantation, Infantile Krabbe disease, Confirmatory testing, Galactocerebrosidase, Lysosomal storage disorder, Psychosine

## Abstract

**Background:**

Krabbe disease is a rare neurodegenerative genetic disorder caused by deficiency of galactocerebrosidase. Patients with the infantile form of Krabbe disease can be treated at a presymptomatic stage with human stem cell transplantation which improves survival and clinical outcomes. However, without a family history, most cases of infantile Krabbe disease present after onset of symptoms and are ineligible for transplantation. In 2006, New York began screening newborns for Krabbe disease to identify presymptomatic cases. To ensure that those identified with infantile disease received timely treatment, New York public health and medical systems took steps to accurately diagnose and rapidly refer infants for human stem cell transplantation within the first few weeks of life. After 11 years of active screening in New York and the introduction of Krabbe disease newborn screening in other states, new information has been gained which can inform the design of newborn screening programs to improve infantile Krabbe disease outcomes.

**Findings:**

Recent information relevant to Krabbe disease screening, diagnosis, and treatment were assessed by a diverse group of public health, medical, and advocacy professionals. Outcomes after newborn screening may improve if treatment for infantile disease is initiated before 30 days of life. Newer laboratory screening and diagnostic tools can improve the speed and specificity of diagnosis and help facilitate this early referral. Given the rarity of Krabbe disease, most recommendations were based on case series or expert opinion.

**Conclusion:**

This report updates recommendations for Krabbe disease newborn screening to improve the timeliness of diagnosis and treatment of infantile Krabbe disease. In the United States, several states have begun or are considering Krabbe disease newborn screening. These recommendations can guide public health laboratories on methodologies for screening and inform clinicians about the need to promptly diagnose and treat infantile Krabbe disease. The timing of the initial referral after newborn screening, the speed of diagnostic confirmation of infantile disease, and the transplantation center’s experience and ability to rapidly respond to a suspected patient with newly diagnosed infantile Krabbe disease are critical for optimal outcomes.

## Background

Krabbe disease (KD) is an autosomal recessive, neurodegenerative disease caused by deficiency of the lysosomal enzyme galactocerebrosidase (GALC), which is essential for myelin turnover [1] and is encoded by the *GALC* gene. In the infantile form of KD (IKD), children can appear normal at birth, but in the first year, usually the first months, they develop irritability, feeding difficulties, progressive spasticity, blindness and deafness. Over time, IKD patients cease to have voluntary movements, and death occurs in infancy or childhood [2]. Before New York (NY) instituted newborn screening for KD in 2006 [2], the estimated incidence was thought to be about 1 in 100,000 births [1, 2], with the majority of KD patients expected to have IKD. The most common *GALC* mutation seen in IKD patients of European ancestry is a 30-kb deletion starting at intron 10 (of the 17-exon gene) and extending beyond the end of the gene. In 2004, Gelb and colleagues described a high-throughput GALC enzyme assay making use of dried blood spots (DBS) [3], and in 2005, Escolar and colleagues reported that presymptomatic human stem cell transplantation (HSCT) in IKD resulted in greatly improved outcomes compared to those who were untreated or treated after symptoms began [4].

Given the potential benefits of HSCT in presymptomatic infants with IKD, NY became the first state to mandate and implement KD-NBS to enable early diagnosis and treatment of KD. The experience of the first 8 years of newborn screening has recently been described [5, 6]. The incidence of IKD in NY was lower than expected with only five affected infants (including one sibling pair) identified among nearly two million screened (1/394,000) [5]. Only four infants ultimately received HSCT (the family of one infant with IKD initially refused, but they agreed to HSCT for a later-born sibling) and in this small cohort, two died and one had severe developmental delays [6]. The NY experience emphasizes the challenges inherent in treating IKD, where symptoms appear so early and progress so quickly that HSCT, to be done at a “presymptomatic stage,” needs to be initiated in the first month of life, and may be, even then, too late [6].

The NY outcomes were unexpectedly poor given what was known of the previously reported cohort transplanted at Duke University [4] and at other sites [7] where combined mortality was 10%. For example, only 1 of the 5 infants in NY was referred to a specialized transplant center in time to have HSCT before 30 days of age [6]. A recent report of the long-term developmental outcomes of 18 IKD infants who were transplanted presymptomatically at less than 2 months of age, showed that the 10 who were transplanted in the first 4 weeks of life had better survival and daily function [8]. This suggests that IKD patients identified by NBS might have better outcomes if they are transplanted in the first month of life at a HSCT center experienced with this disorder.

The challenging timeline needed to improve IKD outcomes was the impetus for establishing a multi-state and multi-disciplinary KD-NBS task force (“Task Force”) to review the literature, share recent experiences and develop new guidance to improve the speed of IKD diagnosis, and initiation of HSCT.

## Methods

The Task Force members (including all co-authors) were drawn from a larger group of public health, medical, and advocacy professionals who met in person in October 2015 to discuss how IKD outcomes might be improved after KD-NBS. The Task Force met by phone and in-person meetings between October 2015 and July 2017 to discuss the existing evidence, from which consensus recommendations were developed.

**Fig. 1:**
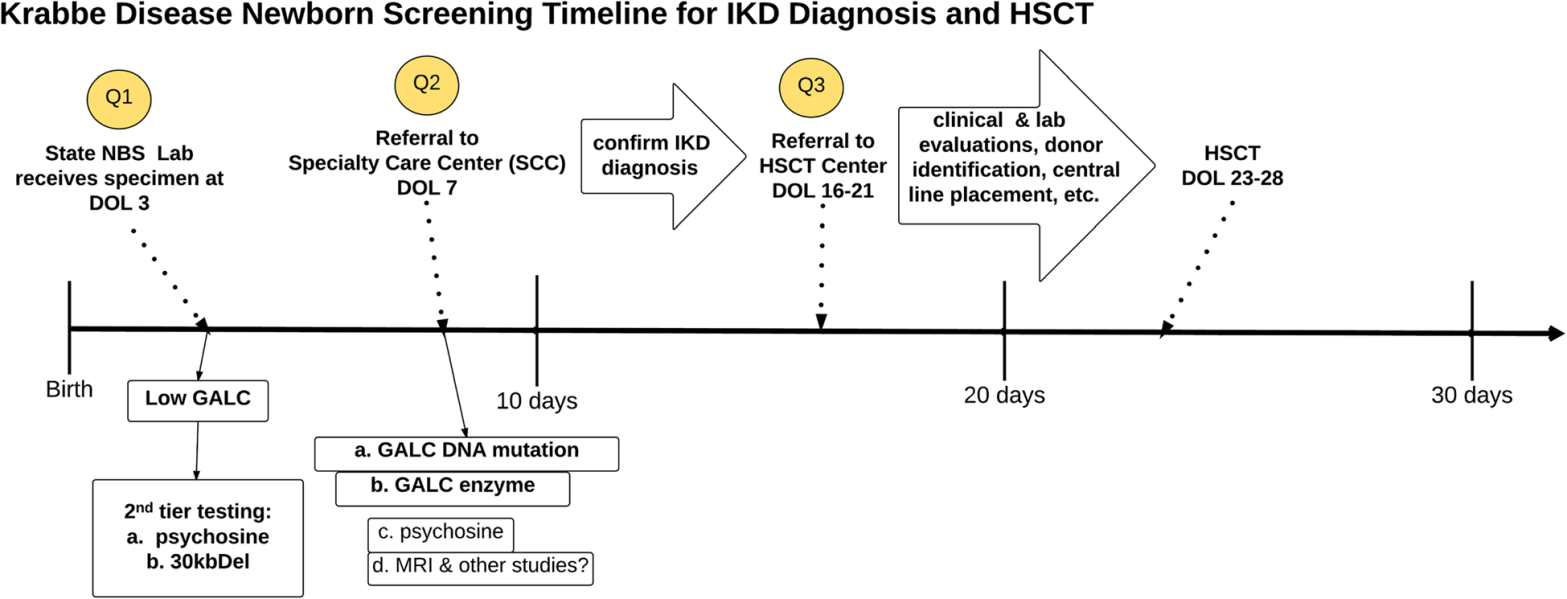
Recommended flow of KD-NBS with optimal times of events, such as receipt of specimen and referral to specialty care center, indicated by number of days of life. The labels, “Q1-Q3,” highlight the key questions 1-3 (see text) being addressed in this evidence review. DOL is infant age in days of life. Additional figure abbreviations: NBS = newborn screening; HSCT = human stem cell transplantation

Figure 1 shows a simplified graph of ideal time-points for KD-NBS, diagnosis of IKD, and referral to HSCT program. The graph also shows the key questions (labelled Q1-Q3 in Fig. 1) being addressed by this Task Force.

### Key questions to be addressed (Fig. 1)

**Question 1.** Newborn Screening Laboratory: Are there preferred laboratory methods and workflows to ensure acceptable sensitivity, specificity, and timeliness in identifying IKD patients?

- Which methods are recommended as the primary screening test for IKD in DBS?
- Is second tier testing (subsequent studies for additional, more specific disease markers using the original NBS DBS sample) necessary? If so, which tests should be considered?
- How quickly does the laboratory need to report a positive screening result for IKD?

**Question 2.** Specialty Care Center (SCC): After the laboratory makes a referral, what confirmatory diagnostic testing should be performed to establish an IKD diagnosis?

- What testing is necessary to establish a diagnosis of IKD?
- Should ancillary neurodiagnostic tests such as from cerebrospinal fluid (CSF), magnetic resonance imaging (MRI), or electromyography, be obtained?

**Question 3.** Treatment: What criteria should be considered when referring IKD patients for HSCT?

Sources of Evidence: For the questions above, the Task Force collected available evidence in the form of 1) relevant articles identified by literature search of MEDLINE, EMBASE, and CINAHL databases, and 2) unpublished data provided by experts which were put in written abstract format and reviewed by members of the work group. The evidence review procedures were based on the Scottish Intercollegiate Guidelines Network (SIGN) criteria [9, 10] used in guideline development for other rare disorders [11, 12]. Relevant articles and data were judged on their evidence quality. Level 1 evidence was based on randomized controlled trials; 2 on case-control or cohort studies; 3 on case series or reports; 4 on expert opinion. Recommendations in response to the questions above were graded A-D based on evidence quality, with grade A recommendations based primarily on high-quality level 1 evidence; B on levels 1 and 2 evidence; C on high quality case-control and cohort studies; and D on biased case-control studies, non-analytic case series, case reports or expert opinion.

## Results

### Question 1: Newborn screening for Krabbe disease using DBS

#### Initial screening tests

There are several approaches to DBS GALC enzyme evaluation for KD screening (Table 1). To date, all but one state program uses tandem mass spectrometry (MS/MS) based assays, and several programs screen for several lysosomal enzymes simultaneously [13–16]. Missouri (MO) is currently employing fluorometry to screen for GALC activity and four other lysosomal enzymes [17]. Regardless of the methods, measures of GALC activity alone do not have sufficient specificity; significant overlap exists between GALC activities observed in KD patients, *GALC* mutation carriers and healthy individuals with genotypes conferring in vitro GALC deficiency (pseudo-deficiency). For this reason, KD-NBS programs will typically employ additional tiers of screening if DBS GALC activity is reduced (Table 2).

**Table 1:** DBS based GALC enzyme assays for KD-NBS

Assay Platform	MS/MS	MS/MS	Fluorometry
Marker	GALC activity	GALC activity	GALC activity
Substrate	Synthetic analog of galactosylceramide containing a C8-fatty acyl chain; after incubation with GALC, releases novel ceramide	Synthetic analog of galactosylceramide containing a C7-fatty acyl chain; after incubation with GALC, releases novel ceramide	Artificial fluorogenic compound; after incubation with GALC, releases 4-MU analog that is measured fluorometrically
States using assay	-*	IL, KY, NY, OH	MO
Reference	[3, 39]	[3, 16, 40]	[17]

*Reagents have been discontinued

**Table 2:** Second tier testing methods in KD NBS

Test Method	Rationale	Advantages	Disadvantages
30 kb deletion testing	Known pathogenic mutation, common in IKD patients	Low complexity, rapid assay. When found homozygous indicates IKD.	Rare mutation, whose presence is more likely to indicate carrier status (i.e. “false positive”) and where the absence will still not avoid possible IKD (“false negative”)
Psychosine testing	Appears to be associated with active disease in KD patients	Rapid test that when elevated indicates IKD.	Requires MS/MS equipment with higher sensitivity than that typically used in NBS labs but testing can be regionalized while still ensuring rapid turnaround time.
*GALC* Genotyping	With 30kbDel, it is traditionally considered the “gold standard” 2nd tier testing in KD-NBS, but there may still be GALC deletions missed, leading to false negative results.	Can identify those infants at highest risk for IKD. Provides some reassurance to those who are carriers, have only enzyme lowering polymorphisms, or known “mild” mutations.	Instrumentation and expertise required are beyond the capabilities of most NBS labs. Many GALC mutations identified through KD-NBS are of uncertain clinical significance. Database of all known KD genotypes not available to support genotype interpretations.

#### Second tier screening tests

The strategy for additional testing of samples with low GALC activity in NY has been reported [5, 13]. First, testing of other lysosomal enzymes is performed as a reference to assess sample quality. If there is still concern, full Sanger sequencing of all exons and all exon/intron boundaries of the *GALC* gene, as well as special PCR analysis, “GAP-PCR,” to detect the common 30kbDel serves as the 2nd tier test. This has improved specificity so that infants with low GALC activity and known benign variants are not referred for follow-up [5]. The genotype information can provide additional diagnostic and prognostic information before the infant is referred and can be valuable to the specialists charged with evaluating, providing counselling, and obtaining confirmatory testing. However, few programs have the ability to perform this level of comprehensive molecular genetic testing and genotypes of uncertain significance are frequently encountered, as seen in the ethnically diverse NY population [5, 6].

The MO NBS program has chosen to test only for the pathogenic 30kbDel mutation in those whose initial DBS GALC activity falls below a predetermined threshold. To further reduce the likelihood of false negative results, MO uses a second GALC activity value in their screening which is lower than the first, such that all infants falling below this value are referred to specialty care centers even if the 30kbDel is not detected [17].

Other strategies incorporate measurement of psychosine using liquid chromatography MS/MS as a 2nd tier test [16]. Psychosine is one of several substrates of the GALC enzyme and its accumulation may cause or contribute to the demyelination and neurodegeneration in KD patients. There has been accumulating evidence that measurement of psychosine concentrations in DBS correlates with clinical disease in IKD patients. Psychosine is normal in controls or those with benign *GALC* variants, but elevated in the newborn DBS of KD patients with infantile and symptomatic late onset disease [18–20]. In NY, all confirmed IKD patients that were referred for HSCT had highly elevated psychosine values (Table 3), and psychosine appears to be specific for severe symptomatic KD. In NY, the NBS laboratory has recently added psychosine testing as another second tier test used in conjunction with full Sanger sequencing and 30kbDel detection. NY state’s current approach may be more than necessary for KD-NBS, since the available data from those known to have KD suggest that psychosine appears to be at least as good as standard molecular *GALC* testing in determining the likelihood of IKD [16, 19].

**Table 3:** Summary of previously reported data from NY^a^ comparing IKD infants’ diagnostic results and outcomes to the 8 considered at high risk to develop KD (but who are asymptomatic to date)

	Patient^a^	*GALC* mutations (simplified, allele1//allele2)^a^	WBC GALC^a^(nmol/h/mg)	psychosine^b^ (nmol/L)	Age at HSCT^a^	HSCT Center^c^	Outcome^a^
IKD	1	30kbDel//p.I546T + p.X670Qext*42	0.01	28.0	32 days	A	Alive, significant delays but interactive
	2	30kbDel//30kbDel	0.05	32.2	31 days	A	Death
	3	30kbDel//30kbDel	0.02	38.1	refused	–	Death
	4	30kbDel// p.G360Dfs*2	0.12	60	41 days	B	Alive, severe delays, minimally interactive
	5	30kbDel//30kbDel	0.05	53.1	24 days	B	Death
High risk for KD^a^	*N* = 8	Bi-allelic pathogenic GALC mutations	Range: 0.03-0.12	Range: 0.21-2.7	Currently, all continue to do well and have had no symptoms requiring additional referrals (follow-up ranging from 1 to 9 years, J. Orsini, personal communication)		

^a^See articles on the NY state experience with KD NBS [5, 6] for more detailed information

^b^Psychosine values reported separately by Escolar et al. [20]; assignment of psychosine values to appropriate infant performed by J. Orsini

^c^HSCT Center: “A” centers have 5 or more years or experience with HSCT in young children with inherited metabolic conditions, and they have transplanted at least one patient with presymptomatic IKD in 5 years. Other HSCT programs are labelled “B”

In Kentucky (KY), the NBS program includes KD and two other lysosomal storage diseases (Pompe disease and Mucopolysaccharidosis type I), with three other lysosomal enzymes being tested as reference enzymes [16]. Measuring activity with multiple lysosomal enzymes can increase clinical specificity, especially when these values are used with recently developed postanalytical tools that use variables, such as age at sample collection, birth weight and gestational age [15, 21, 22]. In KY, 2nd tier testing is employed when postanalytical multivariate analysis of the initial DBS LSD enzyme activities are abnormal. The 2nd tier tests include both the measurement of psychosine and testing for the 30kbDel. Full *GALC* sequencing is also performed but only when the post-analytical score is highly suggestive of KD, psychosine is normal, and one copy of 30kbDel is detected. A repeat DBS sample is requested when the post-analytical score is highly suggestive of KD but psychosine is normal and the 30kbDel is not identified [16]. This approach can efficiently and accurately identify newborns with IKD when psychosine is elevated and/or there is 30kbDel homozygosity. Assuming samples are collected on the 2nd day of life and arrive at the NBS laboratory on the following day, this approach allows reporting of abnormal KD-NBS results by the 5th day of life, the recommended age for reporting of abnormal results for critical NBS conditions [23, 24]. When psychosine is normal and only one copy of 30kbDel is present, *GALC* sequencing of KY newborns is performed in a less emergent fashion to rule out later onset KD variants.

A recent case of IKD diagnosed by NBS in KY [16] highlights the value of using psychosine as a second tier test. In this infant, GALC activity was reduced and psychosine was elevated (61 nmol/L; abnormal > 10 nmol/L), prompting expedited referral to a transplant center on the 6th day of life followed by HSCT on the 24th day of life. This infant’s GALC genotyping showed only one pathogenic mutation on standard sequencing and only with further investigation was a novel deletion detected by using comparative genomic hybridization array testing. While the NY NBS laboratory would have identified the deletion, not all screening laboratories have this capability, and the fact that the genotype had not been observed in known IKD patients and in the absence of psychosine measurement, follow up would likely have led to a later transplant initiation. This case suggests an advantage of psychosine measurement over even sophisticated molecular genetic 2nd tier tests for IKD.

**Addressing Question 1: Are there preferred laboratory methods and workflows to ensure acceptable sensitivity, specificity, and timeliness in identifying IKD patients?**

1a. Recommendation: Both MS/MS or fluorometric methods of measuring GALC activity can be used as the primary screen for KD, but neither is sufficiently specific for KD, let alone IKD, to be used alone. (Note: this recommendation was not graded because methodologies employed in NBS laboratories are subject to laboratory quality standards and regulations).

1b. Recommendation: Second tier testing should be done to improve the specificity of screening and the speed in identifying IKD (Table 2). Once a sample is flagged because of an abnormal primary screen, the test that has the highest likelihood of identifying IKD cases using DBS are psychosine analysis with or without subsequent comprehensive molecular genetic analysis of the GALC gene. (Grade C recommendation).

1c. Recommendation: IKD can progress rapidly and must be considered a time-critical condition similar to galactosemia [23, 24]. It is likely that IKD outcomes are better when potential IKD cases are identified early by second tier testing and then referred to SCC’s by the 5-7th day of life (Fig. 1). Depending on the second tier tests chosen, urgent referrals may be initiated if psychosine levels are elevated or if there is a 30kbDel. SCC medical specialists should promptly see these potential IKD cases and have procedures in place for rapid referral to HSCT centers for further evaluation and treatment (see below, especially responses to Questions 2b and 3). (Grade D evidence.)

Of note, these guidelines are meant to ensure more rapid referral of potential IKD cases. The majority of those newborns with out-of-range results on first tier testing will NOT have IKD, but may be at risk for later onset forms of KD. The screening and follow-up protocols in these patients are not “time-critical” and are beyond the scope of this review.

### Question 2: Confirmatory diagnostic testing of newborns referred after abnormal KD-NBS

There are two diagnostic tests commonly used when infants are referred to specialty care centers for confirmatory testing:

- Leukocyte GALC enzyme activity: When NYS began KD-NBS, leukocyte GALC activity, as performed in the Thomas Jefferson Lysosomal Diseases Testing Laboratory directed by Dr. David Wenger, was used as a confirmatory diagnostic assay and low GALC enzyme activity (previously set at ≤0.15 nmol/h/mg protein [2]), from this laboratory was thought to be predictive of who would develop IKD. These infants were categorized as at “high risk” for developing IKD. All 5 IKD patients identified in NYS were in this high-risk category; however, another 8 infants were also in this “high risk” group but they had more reassuring GALC genotypes and – as determined retrospectively - psychosine was not elevated (Table 3) [18, 20]. All 8, at the time of publication, appear to be developing normally [5, 6]. Therefore, leukocyte GALC enzyme activity alone is not specific enough to identify IKD. More sensitive GALC activity assays have been proposed to better discriminate very low GALC activity indicative of IKD [25]. Until such assays become clinically available, psychosine testing in blood (see above) may be useful in the confirmatory testing phase after an abnormal NBS result [19, 20].
- *GALC* genotyping: The NY KD-NBS experience suggests that *GALC* genotype can be useful in identifying those infants likely to develop IKD, since all these infants had biallelic *GALC* mutations which had either been previously associated with IKD or predicted to be deleterious, i.e., frameshift mutations, in-frame deletions, and splice-site mutations [5]. Psychosine concentrations were elevated in the newborn DBS of these 5 IKD cases [20, 21]. The infants with very low leukocyte GALC enzyme activity but normal psychosine (see above) who have not developed IKD have at least one mutation previously seen in a later-onset case [1, 5] or predicted to be “mild” (often missense mutations) (Table 3) [18–20].

Finally, GALC genotyping is limited in its ability to detect GALC gene deletions [26], and overall, generalizations about prognosis can be difficult to make from genotype alone.

As discussed above, there are data indicating that an elevated blood concentration of psychosine is consistent with IKD [18–20]. Accordingly, psychosine testing may have diagnostic value at least when it is found to be elevated. To date, however, psychosine testing has not been included in routine follow up of at-risk patients identified through KD-NBS. Furthermore, longitudinal studies are required to determine if psychosine has value as a biomarker for determining if HSCT should be initiated in patients at risk for late onset KD.

In NY, additional neurodiagnostic studies were included in the protocol for confirmatory testing [2] at the SCC: MRI, lumbar puncture to obtain CSF, and nerve electrophysiology (e.g., nerve conduction studies). While it is known that in symptomatic Krabbe disease, there are MRI white matter changes, elevation in cerebrospinal fluid protein, and abnormal nerve conduction studies, these studies are difficult to interpret [27–30] in the young infant and take time to perform, further delaying referral for HSCT. Furthermore, HSCT centers experienced in performing metabolic stem cell transplants have the resources in place to rapidly perform high quality diagnostic testing. These centers often prefer to do these studies at their center to facilitate rapid interpretation. Therefore, though these neurodiagnostic studies can help resolve questions about risk of IKD, it is more important that steps be taken to refer potential IKD cases to HSCT centers as soon as possible where these studies can be obtained in parallel with other preparations for a possibly necessary transplant.

**Addressing Question 2: What recommendations can be made about confirmatory testing strategies?**

2a. Recommendation: IKD diagnosis traditionally has relied on both GALC activity and GALC mutational testing (with parental phase confirmation). Psychosine testing can aid in decision making, and because it can provide rapid results, it should be done by the SCC if it has not been done earlier. A mechanism must be in place to guarantee accelerated turn-around time for these three laboratory tests—GALC enzyme activity, GALC mutational analysis, and psychosine testing—because of their importance in determining the risk of true IKD and the urgency of HSCT. (Grade C).

2b. Recommendation: Since HSCT centers perform neurodiagnostic studies, such as MRI, lumbar puncture for CSF protein, and nerve conduction studies, when evaluating an infant at risk for IKD, the SCC does not need to perform these studies when assessing IKD risk. (Grade D).

### Question 3. Selection of HSCT centers and timely referral

HSCT can arrest progression of KD through engraftment of donor-derived, enzyme-producing cells in the bone marrow, brain, and other organs [1, 4]. Case series show that HSCT is effective in improving survival and neurologic outcomes in IKD when treatment is started presymptomatically [4, 31, 32]; in these studies, the IKD diagnosis was established before the 2nd week of life (even prenatally). Data on long-term outcomes are limited in this population. Of the two surviving and transplanted IKD patients identified through NY’s KD-NBS program who were transplanted at 31 and 41 days of life, respectively; both have significant neurologic deficits [6]. A recent report of IKD patients transplanted in the first 2 months of life, suggests those transplanted during their first 4 weeks of life did better in terms of their overall survival and function (walking, need for G-tube, among others) than those transplanted during their 2nd month of life [8].

Initiating HSCT in an infant diagnosed by NBS with IKD before 4 weeks of age is challenging. In NY, where the specialists already have genotyping information available at the time they see the patient, the referral from the NBS laboratory may still take over a week due to delays in sampling and shipping. Additional days are spent scheduling the specialist appointment and sending confirmatory testing, by which time the infant may be 2 weeks old. Table 4 shows the tasks of a metabolic transplant center preparing an infant with IKD for umbilical cord blood transplant which could easily take another 2 weeks. To prevent poor IKD treatment outcomes from delays in HSCT, the timing of all referrals needs to be closely scrutinized and streamlined. The patient with IKD identified through KD-NBS in KY demonstrates that more rapid treatment initiation is possible [16].

**Table 4:** Schedule of HSCT Center tasks for infants with IKD requiring HSCT. These are the steps to be taken after: 1) KD-NBS and confirmatory testing established a diagnosis of IKD, 2) diagnosis and care options were discussed with the family

1. Refer to transplant center ASAP (DOL 5-6) (Fig. 1)
2. HSCT Center helps to arrange insurance coverage, lodging, admission for work up
3.Baby admitted to HSCT Center (DOL 7-8):
a. Blood drawn for stat HLA typing (high resolution Class I ABC, Class II DRB1), and studies, including, blood type, and psychosine
b. Maternal blood for donor screening tests
c. CSF for protein, cell count
d. Neuroimaging tests: MRI brain with DTI
e. Neurophysiological tests: EEG, BAER, VEP, nerve conduction tests
f. Neurology and neurodevelopmental consult
g. Hearing and vision evaluations
h. Echocardiogram to check for PFO or PDA. If present, filter IV lines to prevent air emboli
i. Physical therapy consultation
j. When HLA typing is available, search for an unrelated cord blood unit donor, select units (> 4/6 match and > 5×10e7 cells/kg for HLA-confirmatory typing and GAL-C enzyme levels) to be used for final unit selection
k. Proceed with insurance/third party payer authorization for transplantation
l. Place central line and consider G-Tube placement for supplemental feeding
m. Administer chemotherapy (currently 9 days)
n. Make final cord blood unit selection during chemotherapy
o. Administer transplant (DOL 21+)

As with any other highly specialized medical procedure, the best results and fewest complications are achieved at centers that perform the procedure more frequently. Given the rarity of IKD, outcomes are likely to be more variable at less experienced HSCT centers even though these may be geographically closer to the IKD patient (Table 3). For rare disorders like IKD, the number of HSCT centers with experience in treating KD will be small, and IKD families may be presented with significant geographic and financial barriers to receiving timely HSCT at one of these centers. This is a serious consideration when implementing KD-NBS. State programs must have protocols in place to ensure that infants diagnosed with IKD will be referred to an experienced HSCT center that is prepared to respond rapidly.

The goal in making these recommendations is to make sure that the family knows of an IKD diagnosis at a time when reasonable choices can be made. Accordingly, the providers in the SCC not only must quickly establish a probable IKD diagnosis, they also must counsel families of newborns of all therapeutic options, including refusal of HSCT.

**Addressing Question 3: Given that HSCT treatment is effective in improving survival and neurologic outcomes in those with IKD, how can treatment outcomes be optimized?**

3. Recommendation: Expert opinion suggests prompt referral to a center experienced with KD and other metabolic transplants could reduce variability in outcomes. This referral should occur no later than during the 3rd week of life (Fig. 1) to ensure initiation of HSCT during the first 4 weeks of life. To accomplish this, the SCC initially assessing the referred infant should already have in place a clear protocol for rapid referral to an HSCT center. This assumes that preparations between SCC and HSCT centers have been made in anticipation of such a referral, with the goal of minimizing time to initiation of HSCT once a diagnosis of IKD has been established. (Grade D).

## Discussion

KD-NBS remains controversial and there is still much to learn about the full range of disease presentation and management [33, 34]. Dimmock, in a recent commentary cites the poor outcomes after HSCT in NY IKD patients as one reason to reject KD-NBS [35]. In NY only 4 patients with IKD were treated with HSCT [6], and while their outcomes were poorer than prior trials would have suggested [4], the NY IKD cohort was quite small. Case series of transplanted IKD patients suggest that better outcomes might be expected if the diagnosis of IKD was made very early [8] to allow HSCT in the first month of life. These IKD patients were identified presymptomatically because of their family history, and this knowledge allows families and medical providers valuable time to prepare for treatment. Achieving such early diagnosis and treatment is clearly more challenging after newborn screening, yet these burdens have not been insurmountable. Recently, two children were transferred (by plane provided by medical aid service) to Duke University from states (not NY) in order to receive their transplantation as quickly as possible (J. Kurtzberg, personal communication). Insurance coverage has not been a barrier (J. Kurtzberg, personal communication) since IKD is rare (based on NY data, incidence is 1/394,000), and very few centers have the expertise to perform HSCT in such young Krabbe patients.

If the goal of NBS is to screen for disorders where early diagnosis and treatment can significantly change outcomes, then there was reason to think that IKD meets this standard. KD-NBS can potentially identify IKD cases at an age where initiation of HSCT can markedly improve the survival and quality of life of children with IKD. This treatment currently cannot be considered a cure and disability is common [4, 8]. This guidance suggests that outcomes may be improved if greater awareness and efficiencies are introduced at the level of the screening laboratory, the SCC, and the HSCT center. All screening laboratories should have mechanisms for 2nd tier testing (psychosine measurement or some combination of psychosine testing, *GALC* genotyping, and 30kbDel testing) in place to rapidly identify the rare infants with low GALC activity on DBS who are likely to develop IKD. Furthermore, results of the 2nd tier testing should be available by the end of the first week of life. If the SCC clinicians responsible for confirmatory testing can receive this information early in the infant’s 2nd week of life, this allows time to counsel the affected family about the disease, treatment options and possible outcomes, and to discuss the case with the HSCT center so that a coordinated approach to confirmatory diagnostic testing can be planned if the family chose to pursue a transplant. This early notification also gives the HSCT center time to arrange for transfer and identify an appropriate stem cell donor. The choice of HSCT center is also important and there should be a transparent discussion of the preferred HSCT center(s) to be used whenever KD-NBS is considered. These expectations and this timeline places significant burdens on the newborn screening program.

We recognize that given the rarity of IKD, there are only limited data to support these urgent recommendations. We have relied heavily on the opinions of experts directly involved in the screening, diagnosis and treatment of IKD. These opinions and the recent literature about KD-NBS does not constitute a high level of evidence, but this is often the case with rare metabolic disorders [36] These recommendations are meant to help states or regions considering KD-NBS to understand the planning, cooperation, and resources that need to be put in place for successful implementation.

This review has not touched on the issue of those infants who, on confirmatory testing do not have IKD but based on their low GALC enzyme activity and the presence of two GALC mutations are presumed to be at risk for KD, later in life. Counselling the families of those identified with late-onset KD has been extremely challenging in NY, the state that has had the longest experience with KD-NBS. In NY, none of approximately 40 individuals at risk for late-onset KD (with median followup of 5 years) have been reported as having any concerning neurologic symptoms (J. Orsini, personal communication). The lack of reported late-onset cases identified by KD-NBS, the documented variability of late-onset KD progression, and the limited information about the effectiveness of HSCT in this population, have contributed to the reluctance of families to come for routine clinical follow-up [5, 12]. This situation may change as more is learned about HSCT outcomes in late-onset KD and the natural history of late-onset KD. But until then, these guidelines view the primary goal of KD-NBS as identifying IKD as the “core condition,” with the late-onset KD cases considered “secondary targets” or disorders that can be detected in the screening for core panel conditions [37, 38].

The guidance in this review describes a recommended response to a possible diagnosis of IKD. The reported outcomes of IKD patients identified by KD-NBS may improve in the future if KD-NBS programs stress timely identification of IKD patients and pay attention to maximizing efficiency at every stage of the referral process. NBS programs that add KD to their panels must engage their state’s specialty care centers, clinical reference laboratories, HSCT centers and insurers prior to NBS program implementation in order to define the goal of screening and then develop a plan that ensures smooth referral of patients and samples without delays. In addition, long-term follow up programs, including a nationwide and accessible registry, should be established to more quickly gather a robust data set to support future evidence based program adjustments.

## Conclusions

Key questions about KD-NBS and IKD were addressed by evaluating new evidence. One recommendation was that newborn screening laboratories using GALC enzyme activity to screen for KD also employ 2nd tier testing to improve the speed and specificity of making an IKD diagnosis. Screening workflows should be designed to allow IKD to be identified by the 5th day of life. Another recommendation identified confirmatory testing strategies but emphasized that if IKD was likely (psychosine elevated and/or genotype of known pathogenic significance), referral to the HSCT center should be expedited, even if confirmatory tests were pending or would have to be conducted at the HSCT center. The evidence so far indicates that the key to optimal outcomes in IKD is achieving an early diagnosis and prompt initiation of stem cell transplantation. These recommendations will help guide programs considering or currently conducting KD-NBS.

## Abbreviations

30kbDel: 30 kb deletion
CSF: Cerebrospinal fluid
DBS: Dried blood spot
GALC: Galactocerebrosidase
HSCT: Human stem cell transplantation
IKD: Infantile Krabbe disease
KD: Krabbe disease
KD-NBS: Krabbe disease newborn screening
KY: Kentucky (US state)
LSD: Lysosomal storage disorder
MO: Missouri (US state)
MRI: Magnetic resonance imaging
NBS: Newborn screening
NY: New York (US state)
SCC: Specialty Care Center
